# Finding My Voice in Shaping Global and National Dementia Policy: Perspectives from a Person Living with Dementia

**DOI:** 10.1002/alz70860_097731

**Published:** 2025-12-23

**Authors:** Br. John‐Richard Pagan

**Affiliations:** ^1^ Alzheimer's Disease International Lived Experience Panel, Woodbridge, VA, USA; Global Brain Health Institute, Lived Experience Group, Dublin, Ireland; NAPA Advisory Council on Alzheimer's Research, Care, and Services, Washington, DC, USA

## Abstract

I am a passionate dementia advocate living life as an American, Latino, gay, disabled, military veteran, who was diagnosed at age 47 with MCI and at 54 with Lewy Body Dementia. This has caused mild to moderate cognitive impairments in processing, language, and attention. My diagnosis does not define me and I remain active in spiritual and social communities, with a strong focus on family and support.

My presentation will bring the perspective of a person living with dementia to discuss engaging people with lived experience in dementia research and policy. I will illustrate how I “found my voice” as a person living with dementia, invited to contribute to shaping global and national dementia policies. I have the privilege of being a member of three advisory groups: the NAPA Advisory Council on Alzheimer's Research, Care, and Services, a US governmental body which informs dementia policy, research directions, and the national dementia plan; the Global Brain Health Institute Lived Experience Group, which mentors young researchers globally who are Atlantic Fellows for Equity in Brain Health; and the Alzheimer's Disease International Lived Experience Panel which facilitates global discussions regarding dementia policy and advocacy.

I will speak about my perceptions of positive elements of these panels, such as sharing personal experiences and influencing decision‐makers. I will illustrate negative elements that compromise my participation including difficult logistics for a person with cognitive challenges, not being taken seriously, and feeling trivialized and tokenized. I have been supported when the meeting facilitator takes a personal interest and encourages me and have experienced barriers when I am viewed as not having the ability to be creative and contribute.

I will share my own experience with other countries’ policies on how people with lived experience are engaged in dementia research. I will draw lessons from these experiences on how countries can learn from each other about best practices for engaging people with lived experience in dementia policy and research. This will include recommendations on how dementia researchers can learn to better engage with people living with dementia and help them feel welcomed to these panels and activities.

